# Modified artery-first approach for distal pancreatectomy with celiac axis resection

**DOI:** 10.1093/bjsopen/zrad022

**Published:** 2023-05-05

**Authors:** Huan Wang, Shuo Shen, Yiwei Ren, Xueru Lin, Xiaohan Shi, Suizhi Gao, Bo Li, Xiaoyi Yin, Guoxiao Zhang, Wuchao Liu, Jian Wang, Jiawei Han, Lingyu Zhu, Xiongfei Xu, Zhuo Shao, Wei Jing, Bin Song, Peng Cheng, Shiwei Guo, Kailian Zheng, Gang Jin

**Affiliations:** Department of Hepatobiliary Pancreatic Surgery, Changhai Hospital, Navy Medical University (Second Military Medical University), No. 168 Changhai Road, Yangpu District, Shanghai, 200433, China; Department of Hepatobiliary Pancreatic Surgery, Changhai Hospital, Navy Medical University (Second Military Medical University), No. 168 Changhai Road, Yangpu District, Shanghai, 200433, China; Department of Hepatobiliary Pancreatic Surgery, Changhai Hospital, Navy Medical University (Second Military Medical University), No. 168 Changhai Road, Yangpu District, Shanghai, 200433, China; Medical Affairs Office, Teaching and Research Support Centre, Naval Medical University (Second Military Medical University), No. 800 Xiangyin Road, Yangpu District, Shanghai, 200433, China; Department of Hepatobiliary Pancreatic Surgery, Changhai Hospital, Navy Medical University (Second Military Medical University), No. 168 Changhai Road, Yangpu District, Shanghai, 200433, China; Department of General Surgery, Naval Medical Centre of People's Liberation Army (PLA), Naval Medical University (Second Military Medical University), No. 338 West Huaihai Road, Changning District, Shanghai, 200050, China; Department of Hepatobiliary Pancreatic Surgery, Changhai Hospital, Navy Medical University (Second Military Medical University), No. 168 Changhai Road, Yangpu District, Shanghai, 200433, China; Department of General Surgery, Naval Medical Centre of People's Liberation Army (PLA), Naval Medical University (Second Military Medical University), No. 338 West Huaihai Road, Changning District, Shanghai, 200050, China; Department of Hepatobiliary Pancreatic Surgery, Changhai Hospital, Navy Medical University (Second Military Medical University), No. 168 Changhai Road, Yangpu District, Shanghai, 200433, China; Department of Hepatobiliary Pancreatic Surgery, Changhai Hospital, Navy Medical University (Second Military Medical University), No. 168 Changhai Road, Yangpu District, Shanghai, 200433, China; Department of Hepatobiliary Pancreatic Surgery, Changhai Hospital, Navy Medical University (Second Military Medical University), No. 168 Changhai Road, Yangpu District, Shanghai, 200433, China; Department of Hepatobiliary Pancreatic Surgery, Changhai Hospital, Navy Medical University (Second Military Medical University), No. 168 Changhai Road, Yangpu District, Shanghai, 200433, China; Department of Hepatobiliary Pancreatic Surgery, Changhai Hospital, Navy Medical University (Second Military Medical University), No. 168 Changhai Road, Yangpu District, Shanghai, 200433, China; Department of Hepatobiliary Pancreatic Surgery, Changhai Hospital, Navy Medical University (Second Military Medical University), No. 168 Changhai Road, Yangpu District, Shanghai, 200433, China; Department of Hepatobiliary Pancreatic Surgery, Changhai Hospital, Navy Medical University (Second Military Medical University), No. 168 Changhai Road, Yangpu District, Shanghai, 200433, China; Department of Hepatobiliary Pancreatic Surgery, Changhai Hospital, Navy Medical University (Second Military Medical University), No. 168 Changhai Road, Yangpu District, Shanghai, 200433, China; Department of Hepatobiliary Pancreatic Surgery, Changhai Hospital, Navy Medical University (Second Military Medical University), No. 168 Changhai Road, Yangpu District, Shanghai, 200433, China; Department of Hepatobiliary Pancreatic Surgery, Changhai Hospital, Navy Medical University (Second Military Medical University), No. 168 Changhai Road, Yangpu District, Shanghai, 200433, China; Department of Hepatobiliary Pancreatic Surgery, Changhai Hospital, Navy Medical University (Second Military Medical University), No. 168 Changhai Road, Yangpu District, Shanghai, 200433, China; Department of Hepatobiliary Pancreatic Surgery, Changhai Hospital, Navy Medical University (Second Military Medical University), No. 168 Changhai Road, Yangpu District, Shanghai, 200433, China; Department of Hepatobiliary Pancreatic Surgery, Changhai Hospital, Navy Medical University (Second Military Medical University), No. 168 Changhai Road, Yangpu District, Shanghai, 200433, China; Department of Hepatobiliary Pancreatic Surgery, Changhai Hospital, Navy Medical University (Second Military Medical University), No. 168 Changhai Road, Yangpu District, Shanghai, 200433, China

## Abstract

**Background:**

The superior mesenteric artery-first approach has been proved superior in pancreatoduodenectomy compared with the standard procedure. It is unclear whether similar benefits could be obtained in distal pancreatectomy with celiac axis resection.

**Methods:**

Perioperative and survival outcomes of patients who underwent distal pancreatectomy with celiac axis resection with the modified artery-first approach or traditional approach between January 2012 and September 2021 were compared.

**Results:**

The entire cohort comprised 106 patients (modified artery-first approach, *n* = 35; traditional approach, *n* = 71). The most common complication was postoperative pancreatic fistula (*n* = 18, 17.0 per cent), followed by ischaemic complications (*n* = 17, 16.0 per cent) and surgical site infection (*n* = 15, 14.0 per cent). Intraoperative blood loss (400 *versus* 600 ml, *P* = 0.017) and intraoperative transfusion rate (8.6 *versus* 29.6 per cent, *P* = 0.015) were lower in the modified artery-first approach group compared with the traditional approach group. A higher number of harvested lymph nodes (18 *versus* 13, *P* = 0.030) and R0 resection rate (88.6 *versus* 70.4 per cent, *P* = 0.038) and a lower incidence of ischaemic complications (5.7 *versus* 21.1 per cent, *P* = 0.042) was observed in the modified artery-first approach group compared with the traditional approach group. In multivariable analysis, the modified artery-first approach (OR 0.006, 95 per cent c.i., 0 to 0.447; *P* = 0.020) was protective against ischaemic complications.

**Conclusions:**

Compared with the traditional approach, the modified artery-first approach was associated with lower blood loss and fewer ischaemic complications, and a higher number of harvested lymph nodes and R0 resection rate. Thus, it might improve the safety, staging and prognosis of distal pancreatectomy with celiac axis resection for pancreatic cancer.

## Teaser

In the present study, the modified artery-first approach was associated with lower blood loss and fewer ischaemic complications, and a higher number of harvested lymph nodes and R0 resection rate. Thus, it might improve the safety, staging and prognosis of distal pancreatectomy with celiac axis resection for pancreatic cancer.

## Introduction

Pancreatic cancer is an aggressive disease expected to become the second leading cause of cancer death within the next 10 years^1,2^. Surgical resection offers a curative chance for pancreatic cancer patients, but more than 80 per cent of those are not suitable for resection at the time of diagnosis^3^. Lesions of the pancreas body/tail with involvement of the celiac axis (CA) > 180° are considered locally advanced^4^. Those lesions, when the aorta and/or the gastroduodenal artery (GDA) are not involved, can be radically resected through distal pancreatectomy with celiac axis resection (DP-CAR), especially if induction chemotherapy is administrated prior to surgery.

DP-CAR is a challenging procedure. Local radicality is difficult to achieve in locally advanced pancreatic cancer (LAPC) due to significant invasion of retroperitoneal tissues. Previous international multicentre studies reported an R0 resection rate of 54–59 per cent^5,6^. DP-CAR is associated with a high morbidity rate and a 90-day mortality rate as high as 16–18 per cent^5,6^. According to previous studies^6^, ischaemic complications of DP-CAR account for one-third of the 90-day mortality rate and cannot be prevented by existing strategies. Considering these limitations, appropriate preoperative patient selection may avoid DP-CAR in patients who may not benefit from it^6,7^. Similarly, it is crucial to determine whether DP-CAR is applicable in the early phase of the operation.

The superior mesenteric artery-first approach was proposed to determine arterial involvement prior to pancreas transection during pancreatoduodenectomy^8,9^. The same concept can be applied to CA and DP. The aim of the present study is to compare the modified artery-first approach (mAFA) for DP-CAR to the traditional approach (TA) in terms of postoperative and long-term outcomes.

## Methods

### Study population

All patients scheduled for DP-CAR were reviewed at the Changhai Hospital, Naval Military Medical University, between January 2012 and September 2021. Patients’ data was retrospectively retrieved from the Changhai prospective database of pancreatic tumours. The project was approved by the Changhai Hospital Ethics Committee (CHEC2021-140).

### Preoperative evaluation

Preoperative assessments included medical history, physical examination, laboratory testing and contrast-enhanced computed tomography. Since 2016, most patients received at least three cycles of neoadjuvant therapy (NAT), and the NAT regime was decided by the attending doctor. The indications for DP-CAR were discussed in a multidisciplinary meeting and included tumour of the pancreatic body/tail with involvement of the common hepatic artery (CHA) and/or CA, and without involvement of the superior mesenteric artery (SMA), proper hepatic artery (PHA), GDA or distant metastasis.

### Surgical technique

#### Step 1. Exploration and GDA backflow assessment

The abdomen was opened preferably through a midline incision and the absence of distant metastasis was confirmed.

##### mAFA group

**Fig. 1:**
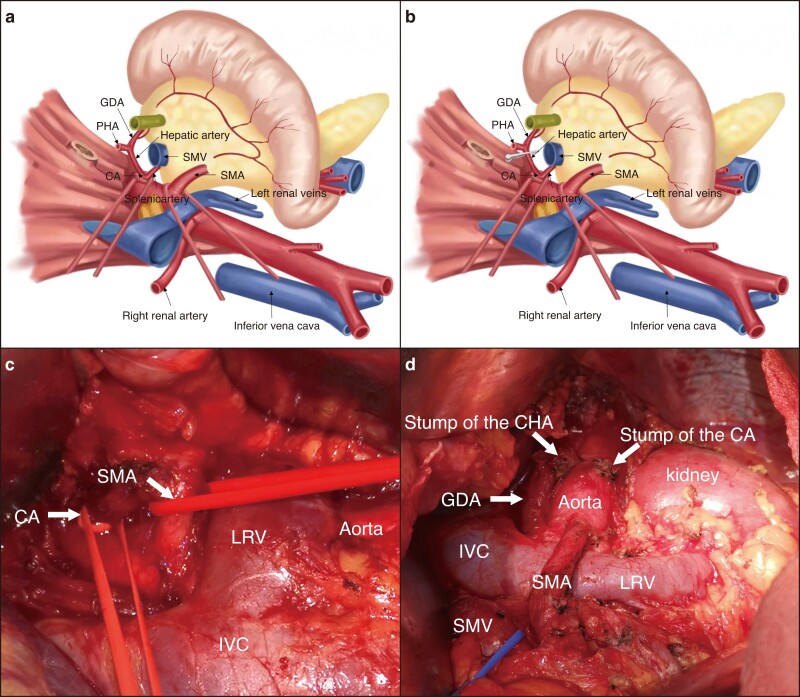
Illustration of the operative field during modified artery-first approach (mAFA) DP-CAR **a**, **c** Operative field after the Kocher manoeuvre. **b** Illustration of the operative field after clamping of the common hepatic artery (CHA) and isolation of the celiac axis (CA). **d** Operative field after mAFA distal pancreatectomy with CA resection. PHA, proper hepatic artery; GDA, gastroduodenal artery; SMA, superior mesenteric artery; SMV, superior mesenteric vein; IVC, inferior vena cava; LRV, left renal vein.

The first step included a wide Kocher’s manoeuvre and dissection of the soft tissues along the CA and SMA. A vessel loop was used to wrap the roots of CA and SMA (*Fig. 1a*, *c*). After clamping the CA root, dissection of the CHA was performed, the CHA was then temporarily clamped (*Fig. 1b*) and the blood flow of the PHA and right gastroepiploic artery (RGA) was assessed using ultrasonography and/or palpation. If the blood flow was considered sufficient, the CHA was ligated. Otherwise, the DP-CAR procedure was abandoned, and radical antegrade modular pancreatosplenectomy (RAMPS)^10^ with arterial divestment or a palliative procedure was performed, according to the severity of arterial infiltration.

##### TA group

TA has been previously described^11,12^. The Kocher's manoeuvre was not performed in this group. The first step included assessment of the backflow in the GDA clamping the CHA. The CA, instead, was isolated only after pancreatic neck transection.

#### Step 2. Pancreatic transection and perivascular clearance

The pancreas was transected using a linear stapler. If the tumour was too close to the bifurcation of GDA and PHA and it was not possible to use a linear stapler, GDA was separated from the pancreas by cold dissection, the pancreas was transected with a scalpel and a hand-sewn closure was used for the pancreatic stump. The splenic vein was dissected and ligated. Superior mesenteric vein (SMV)/portal vein (PV) resection and reconstruction were performed if required. To achieve local radicality, the SMA was skeletonized. CA was ligated at its origin. The soft tissue between SMA, CA and SMV/PV was removed.

#### Step 3. En bloc resection of the tumour and retropancreatic tissues

To perform complete retropancreatic dissection, the RAMPS method^10^ was used. In most cases, the Gerota’s fascia was cut, and the anterior aspect of the para-renal fat and the pancreatic body and tail, spleen and left adrenal gland were resected (*Fig. 1d*). A partial gastrectomy was performed if the stomach was involved.

### Intra- and postoperative data

Surgical outcomes included blood loss volume, blood transfusion rate, and concomitant vein or stomach resection. Pathological data included tumour size, differentiation grade, number of positive lymph nodes and R0 resection rate. *N* staging was classified according to the 8th edition of the AJCC Cancer Staging Manual^13^. R0 resection was defined as a resection margin more than 1 mm away from the tumour^14^.

Postoperative pancreatic fistula (POPF)^15^, delayed gastric emptying (DGE)^16^ and postpancreatectomy haemorrhage (PPH)^17^ were deﬁned according to the deﬁnitions proposed by the International Study Group (ISGPS). Grade B/C DGE was defined as clinically relevant DGE (CR-DGE). The severity of complications was graded using the Clavien–Dindo classiﬁcation^18^. Major complications were defined as grade III or higher. Ischaemic complications included postoperative gastric or liver ischaemia, and other complications caused by insufficient arterial perfusion after DP-CAR. In particular, gastric ischaemia was defined as postoperative gastric dysfunction with endoscopic evidence of ischaemic findings (for example ulceration/necrosis) and/or gastric perforation identified by imaging or during reoperation. Liver ischaemia was defined as elevated alanine aminotransferase (ALT) (more than 500 IU/l) levels within 3 days after surgery. Postoperative liver function was tested on postoperative days 1 and 3. Overall survival (OS) was defined as the time from date of surgery to patient death. Follow-up ended in December 2021.

### Statistical analysis

Data were analysed using SPSS 25.0. Categorical variables were expressed as absolute frequencies and percentages, and compared using the chi-square test or Fisher's exact test. Continuous variables were expressed as median with ranges and compared using the Mann–Whitney *U* test. To identify risk factors for ischaemic complications, preoperative and intraoperative variables were included in univariate/multivariate analyses based on the Akaike information criterion. Survival curves were constructed using the Kaplan–Meier method and assessed by log rank test, which was performed by GraphPad Prism 8.0. *P* < 0.05 were considered statistically significant.

## Results

### Patient population

**Fig. 2:**
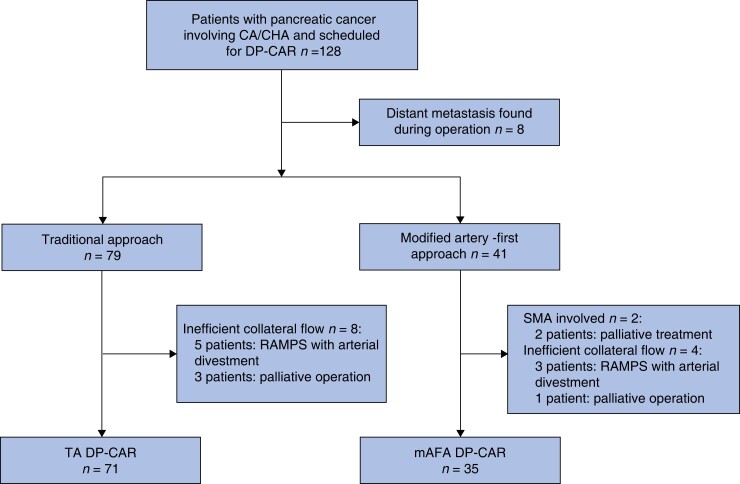
Flow chart of included patients

CA, celiac axis; CHA, common hepatic artery; DP-CAR, distal pancreatectomy with CA resection; SMA, superior mesenteric artery; RAMPS, radical antegrade modular pancreatosplenectomy; TA, traditional approach; mAFA, modified artery-first approach.

A total of 128 consecutive patients with pancreatic cancer of the body/tail involving the CA/CHA scheduled for DP-CAR were included (*Fig. 2*). Eight patients were excluded due to distant metastasis. For eight patients (10.1 per cent) in the TA group and four (9.8 per cent) in the mAFA group, DP-CAR was abandoned due to insufficient blood flow. Two patients (4.9 per cent) in the mAFA group were excluded due to SMA invasion. Overall, 106 patients underwent DP-CAR, 35 in the mAFA group and 71 in the TA group.

*Table 1* shows baseline characteristics. Some 29 patients (27.4 per cent) received preoperative NAT, with most patients receiving gemcitabine/abraxane (*n* = 21, 19.8 per cent). A significantly higher proportion of patients in the mAFA group (*n* = 25, 71.4 per cent) received NAT compared with the TA group (*n* = 4) (71.4 per cent *versus* 5.6 per cent, *P* < 0.001). Preoperatively, carbohydrate antigen 19-9 (CA 19-9; 54.8 *versus* 367.7 U/ml, *P* = 0.011) and carcinoembryonic antigen (CEA; 2.6 *versus* 3.7 U/ml, *P* = 0.035) levels were lower in the mAFA group. Preoperative liver function and routine blood tests were similar between the two groups.

**Table 1:** Baseline characteristics

Variable	All (*n* = 106)	mAFA (*n* = 35)	TA (*n* = 71)	*P*
**Age, median (i.q.r., years)**	64.5 (54.8–70.0)	64.0 (54.0–68.0)	65.0 (55.0–71.0)	0.378
**Sex**				0.537
Male	62 (58.5)	19 (54.3)	43 (60.6)	
Female	44 (41.5)	16 (45.7)	28 (39.4)	
**BMI, median (i.q.r., kg/m^2^)**	22.9 (20.2–24.3)	22.8 (20.9–24.4)	22.9 (19.7–24.3)	0.762
**Previous abdominal surgery**	21 (19.8)	9 (25.7)	12 (16.9)	0.284
**Diabetes mellitus**	23 (21.7)	4 (11.4)	19 (26.8)	0.072
**Coronary artery disease**	6 (5.7)	3 (8.6)	3 (4.2)	0.643
**Hypertension**	34 (32.1)	12 (34.3)	22 (31.0)	0.732
**Cerebrovascular disease**	4 (3.8)	2 (5.7)	2 (2.8)	0.846
**Hepatic diseases**	6 (4.7)	3 (8.6)	3 (4.2)	0.643
**Smoking history**	27 (25.5)	11 (31.4)	16 (22.5)	0.323
**Drinking history**	11 (10.4)	5 (14.3)	6 (8.5)	0.557
**Presenting symptoms**				
Abdominal pain	75 (70.8)	25 (71.4)	50 (70.4)	0.915
Dyspepsia	37 (34.9)	12 (34.3)	25 (35.2)	0.925
Back pain	51 (48.1)	20 (57.1)	31 (43.7)	0.191
Weight loss	50 (47.2)	16 (45.7)	33 (47.9)	0.833
Incidental diagnosis	11 (10.4)	4 (11.4)	7 (9.9)	1.000
**Neoadjuvant radiotherapy**	11 (10.4)	10 (28.6)	1 (1.4)	<0.001
**Neoadjuvant chemotherapy**	29 (27.4)	25 (71.4)	4 (5.6)	<0.001
Gemcitabine/abraxane	21 (19.8)	21 (60.0)	0 (0)	
Abraxane/S-1	1 (0.9)	1 (2.9)	0 (0)	
Gemcitabine/S-1	2 (1.9)	1 (2.9)	1 (1.4)	
Gemcitabine/oxaliplatin	3 (2.8)	1 (2.9)	2 (2.8)	
S-1	1 (0.9)	0 (0)	1 (1.4)	
Other	1 (0.9)	1 (2.9)	0 (0)	
**Preoperative CA 19–9, median (i.q.r., U/ml)**	259.7 (23.7–1200.0)	54.8 (9.8–375.3)	367.7 (39.9–1200.0)	0.011
**Preoperative CEA, median (i.q.r., U/ml)**	3.2 (2.0–6.8)	2.6 (1.9–4.2)	3.7 (2.2–7.9)	0.035
**Preoperative ALT, median (i.q.r., U/l)**	17.0 (13.0–25.0)	20.0 (14.0–31.0)	16.0 (12.0–25.0)	0.082
**Preoperative AST, median (i.q.r., U/l)**	18.0 (15.0–20.3)	18.0 (16.0–22.0)	18.0 (15.0–20.0)	0.390

Values are *n* (%) unless otherwise indicated. mAFA, modified artery-first approach; i.q.r., interquartile range; CA19–9, carbohydrate antigen 19-9; CEA, carcinoembryonic antigen; ALT, alanine aminotransferase; AST, aspartate aminotransferase.

### Operative characteristics and pathological findings

The median operative time for the overall cohort was 137.5 min, with no significant difference between the mAFA and TA groups (130 *versus* 140 min, *P* = 0.448). The mAFA group had less intraoperative blood loss compared with the TA group (400 *versus* 600 ml, *P* = 0.017) and the intraoperative transfusion rate was lower (*n* = 3, 8.6 per cent *versus n* = 21, 29.6 per cent, *P* = 0.015). Some 15 (14.2 per cent) underwent SMV/PV resection, while 7 (6.6 per cent) underwent partial gastrectomy (*Table 2*).

**Table 2:** Operative characteristics and pathological findings

Variable	All (*n* = 106)	mAFA (*n* = 35)	TA (*n* = 71)	*P*
**Operative time, median (i.q.r., min)**	137.5 (120.0–165.0)	130.0 (120.0–180.0)	140.0 (115.0–160.0)	0.448
**Blood loss, median (i.q.r., ml)**	500.0 (300.0–800.0)	400.0 (200.0–500.0)	600.0 (300.0–800.0)	0.017
**Blood transfusion**	24 (22.6)	3 (8.6)	21 (29.6)	0.015
**Portal vein resection**	15 (14.2)	6 (17.1)	9 (12.7)	0.746
**Left adrenalectomy**	59 (55.7)	33 (94.3)	26 (36.6)	<0.001
**Partial gastrectomy**	7 (6.6)	3 (8.6)	4 (5.6)	0.875
**Tumour size, median (i.q.r., cm)**	4.2 (3.0–5.5)	3.5 (2.5–6.0)	4.5 (3.0–5.5)	0.078
**Differentiation**				0.449
Poorly differentiated	20 (18.9)	7 (20.0)	19 (26.8)	
Moderately differentiated	86 (81.1)	28 (80.0)	52 (73.2)	
Well differentiated	0 (0)	0 (0)	0 (0)	
**Lymph nodes detected, (i.q.r.)**	14.0 (7.0–21.3)	18.0 (9.0–26.0)	13.0 (5.0–18.0)	0.030
**Lymph node metastasis**				0.188
N0	46 (43.4)	13 (37.1)	33 (46.5)	
N1	44 (41.5)	14 (40.0)	30 (42.3)	
N2	16 (15.1)	8 (22.9)	8 (11.3)	
**Lymphovascular invasion**	26 (24.5)	8 (22.9)	18 (25.4)	0.779
**Perineural invasion**	98 (92.5)	34 (97.1)	64 (90.1)	0.372
**R0 resection**	81 (76.4)	31 (88.6)	50 (70.4)	0.038

Values are *n* (%) unless otherwise indicated. mAFA, modified artery-first approach; i.q.r., interquartile range.

The median tumour diameter was 4.2 cm. All tumours were poorly (*n* = 20, 18.9 per cent) or moderately (*n* = 86, 81.1 per cent) differentiated. A higher number of lymph nodes were harvested in the mAFA group compared with the TA group (18.0 *versus* 13.0, *P* = 0.030). There was no significant difference between the two groups in terms of *N* stage, lymphovascular invasion and perineural invasion. The R0 resection rate was higher in the mAFA group than in the TA group (*n* = 31, 88.6 per cent *versus n* = 50, 70.4 per cent, *P* = 0.038).

### Operative outcomes

The most common complication of DP-CAR was POPF (*n* = 18, 17.0 per cent), followed by ischaemic complications (*n* = 17, 16.0 per cent) and surgical site infections (*n* = 15, 14.2 per cent). Major complications occurred in 13 patients (12.3 per cent), and seven patients (6.6 per cent) were re-admitted to the hospital within 30 days after the operation. The incidence of complications, such as POPF, operative site infection and multiple organ dysfunction syndrome (MODS), was not significantly different between the two groups. Similarly, there was no statistical difference in major complications, reoperation rate and 30-day re-admission rate (*Table 3*).

**Table 3:** Surgical outcomes

Variable	All (*n* = 106)	mAFA (*n* = 35)	TA (*n* = 71)	*P*
**POPF**	18 (17.0)	6 (17.1)	12 (16.9)	0.975
**Operative site infection**	15 (14.2)	5 (14.3)	10 (14.1)	1.000
**CR-DGE**	5 (4.7)	3 (8.6)	2 (2.8)	0.408
**PPH**	4 (3.8)	1 (2.9)	3 (4.2)	1.000
**Gastrointestinal haemorrhage**	3 (2.8)	0 (0)	3 (4.2)	0.541
**Chyle leakage**	5 (4.7)	0 (0)	5 (7.0)	0.262
**Ischaemia complications**	17 (16.0)	2 (5.7)	15 (21.1)	0.042
Gastric ischaemia	2 (1.9)	0 (0)	2 (2.8)	0.808
Liver ischaemia	16 (14.2)	2 (5.7)	14 (19.7)	0.058
**MODS**	3 (2.8)	0 (0)	3 (4.2)	0.541
**Reoperation**	5 (4.7)	2 (5.7)	3 (4.2)	1.000
**Clavien–Dindo grade ≥ III**	13 (12.3)	4 (11.4)	9 (12.7)	1.000
**Total time NGT was inserted, median (i.q.r., days)**	2.0 (2.0–3.0)	2.0 (2.0–3.0)	2.0 (1.0–3.0)	0.731
**Duration of hospital stay, median (i.q.r., days)**	8.0 (7.0–11.0)	7.0 (6.0–10.0)	9.0 (7.0–12.0)	0.060
**Hospital costs, median (i.q.r., euros)**	7091.8 (6 227.6–8 140.4)	7162.3 (6 275.5–8 036.2)	6976.4 (6 158.1–8 195.1)	0.579
**30-day re-admission**	7 (6.6)	2 (5.7)	5 (7.0)	1.000
**90-day mortality rate**	4 (3.8)	0 (0)	4 (5.6)	0.374
**POD1 ALT, median (i.q.r., U/l)**	36.5 (23.0–110.0)	39.0 (24.0–88.0)	33.0 (22.0–147.0)	0.704
**POD1 AST, median (i.q.r., U/l)**	39.5 (27.0–104.3)	45.0 (30.0–79.0)	36.0 (26.0–176.0)	0.506
**POD3 ALT, median (i.q.r., U/l)**	44.5 (19.8–173.0)	47.0 (25.0–194.0)	42.0 (18.0–166.0)	0.719
**POD3 AST, median (i.q.r., U/l)**	32.0 (21.0–97.8)	35.0 (23.0–69.0)	29.0 (20.0–115.0)	0.584

Values are *n* (%) unless otherwise indicated. mAFA, modified artery-first approach; TA, traditional approach; i.q.r., interquartile range; POPF, postoperative pancreatic fistula; CR-DGE, clinically relevant delayed gastric emptying; PPH, postpancreatectomy haemorrhage; MODS, multiple organ dysfunction syndrome; NGT, nasogastric tube; POD, postoperative day; ALT, alanine aminotransferase; AST, aspartate aminotransferase.

Postoperative liver function and routine blood tests were not significantly different between the two groups. The median duration of hospital stay was 8 days, with no significant difference between the groups (7 *versus* 9 days, *P* = 0.060). The two groups had similar hospitalization costs (7162.4 *versus* 6976.4 euros, *P* = 0.579).

Some 17 patients (16.0 per cent) developed postoperative ischaemic complications, 15 of whom had liver ischaemia, one had gastric ischaemia, and one had both liver and gastric ischaemia. In the mAFA group, the incidence of ischaemic complications was lower compared with the TA group (*n* = 2, 5.7 per cent *versus n* = 15, 21.1 per cent, *P* = 0.042). In the entire cohort, four patients (3.8 per cent) died within 90 days, all of whom were in the TA group. Deaths were in all cases directly or indirectly related to the ischaemic complications. One patient died due to MODS secondary to acute liver ischaemia 2 days after the surgery. The second patient developed bile leakage due to liver ischaemia, which aggravated POPF and resulted in fatal bleeding 2 weeks after surgery. The remaining two patients suffered gastric perforation and succumbed to severe sepsis almost 1 month after surgery. Except for the four patients mentioned above, the remaining 13 patients recovered from the ischaemic complications after symptomatic treatment. Univariable and multivariable analyses on factors associated with ischaemic complications are shown in *Table S1*. The mAFA technique, preoperative CA 19-9 and tumour size were associated with postoperative ischaemic complications in the univariable analysis. In the multivariable analysis, the mAFA (OR 0.006, 95 per cent c.i., 0–0.447; *P* = 0.020) was identified as an independent protective factor for ischaemic complications.

#### Long-term outcomes

**Fig. 3:**
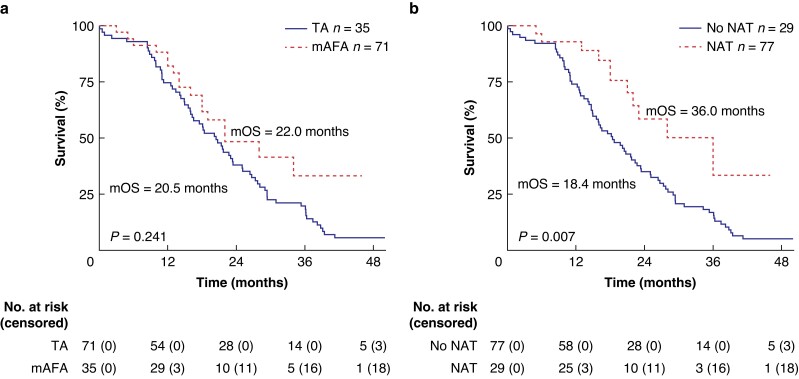
Long-term survival **a** Kaplan–Meier curves comparing the mAFA and TA groups. **b** Kaplan–Meier curves comparing patients with and without preoperative NAT. TA, traditional approach; mAFA, modified artery-first approach; NAT, neoadjuvant therapy; mOS, median overall survival.

The median follow-up was 19.8 months. The median OS was 21.4 months, with no difference between the mAFA and TA groups (22.0 *versus* 20.5 months, *P* = 0.241; *Fig. 3a*). Similarly, there was no significant difference in 1-, 2- and 3-year survival rates between the two groups (1-year survival rate: 88.2 per cent *versus* 74.6 per cent, *P* = 0.079; 2-year survival rate: 48.1 per cent *versus* 38.0 per cent, *P* = 0.269; 3-year survival rate: 30.9 per cent *versus* 19.7 per cent, *P* = 0.272). NAT was associated with a better prognosis compared with non-NAT patients (36.0 *versus* 18.4 months, *P* = 0.007; *Fig. 3b*).

## Discussion

mAFA was associated with lower blood loss, fewer ischaemic complications, and a higher number of harvested lymph nodes and R0 resection rate.

As previously described^19^, massive intraoperative blood loss is a striking feature of DP-CAR, which directly affects its surgical safety, increasing morbidity rate and mortality rate^20,21^. In the mAFA, the CA is clamped at an early phase of the operation, which could explain the lower blood losses described in this study compared with the literature. Ischaemic complications are typical of DP-CAR and they can have severe consequences, such as liver abscess, gastric perforation, sepsis and even death^22^. In the present study, the mAFA group had a lower incidence of ischaemic complications compared with the TA group, and the mAFA was protective against ischaemia in multivariable analysis. Furthermore, four patients in the TA group died of direct or indirect ischaemic complications, while no patient died of ischaemic complications in the mAFA group. This could be explained by an inaccurate assessment of collateral ﬂow in the TA group. Indeed, in this approach, only the CHA is clamped at the beginning, while in the mAFA the CA is clamped at its origin allowing identification of potential perfusion alteration.

For LAPC patients, a typical location for R1 positive margins is in proximity of the SMA and CA^23,24^. In the present study, mAFA achieved a higher rate of R0 resection and detected a higher number of lymph nodes compared with the TA, improving local radicality and quality of DP-CAR. Although the survival analysis showed no significant difference between the groups, this could be explained by the relatively small numbers. It should be noted that a higher percentage of patients in the mAFA group were treated with NAT than the TA group, which may affect the comparison of R0 resection and survival rate between the two groups.

The present study had some limitations. The retrospective nature of the study limits the robustness of the comparison. A 10-year time span might have introduced time-dependent bias. For example, NAT was widely used after 2016 and therefore more patients in the mAFA group underwent NAT. The mAFA described in this study is derived from a high-volume centre with over 1000 pancreatectomies per year, and it is unclear whether similar benefits could also be obtained in low-volume centres. A recent multicentre study^6^ suggested that the 90-day mortality rate in low-volume DP-CAR centres was about three-fold higher than that in high-volume centres (18.0 per cent *versus* 5.5 per cent, *P* = 0.015). Considering the underlying volume-outcome curve for this complex procedure, patients scheduled for DP-CAR should be centralized.

The mAFA technique was proposed to evaluate the root of CA/SMA early in DP-CAR. Clamping of the CA root at the beginning of the operation reduced blood loss and allowed identification of patients at high risk of ischaemic complications. mAFA should be performed in high-volume pancreatic surgery centres to improve safety, staging and prognosis of DP-CAR for pancreatic cancer.

## Supplementary Material

zrad022_Supplementary_Data

## Data Availability

All data generated or analysed during this study are included in this article. Further enquiries can be directed to the corresponding authors.
