# Supplementary material for: Modified artery-first approach for distal pancreatectomy with celiac axis resection
Source: BJS Open. 2023 May 5;7(3):zrad022. doi: 10.1093/bjsopen/zrad022 (PMC10162682; doi:10.1093/bjsopen/zrad022)
Supplement: zrad022_Supplementary_Data [file zrad022_supplementary_data.docx]

**Title:**

Modified artery-first approach for distal pancreatectomy with celiac axis resection: strategy to improve patients’ selection and surgical safety

**Authors**:

Huan Wang^1,*^, Shuo Shen^1,*^, Yiwei Ren^1,*^, Xueru Lin^2,*^,Xiaohan Shi^1,3^, Suizhi Gao^1^, Bo Li^3^, Xiaoyi Yin^1^ ,Guoxiao Zhang^1^, Wuchao Liu^1^, Jian Wang^1^, Jiawei Han^1^, Lingyu Zhu^1^, Xiongfei Xu^1^, Zhuo Shao^1^, Wei Jing^1^, Bin Song^1^, Peng Cheng^1^, Shiwei Guo^1^, Kailian Zheng^1,#^, Gang Jin^1,#^.

**Authors’ Affiliations:**

1. Department of Hepatobiliary Pancreatic Surgery, Changhai Hospital, Naval Medical University (Second Military Medical University), Shanghai, China.

2. Medical Affairs Office, Teaching and Research Support Center, Naval Medical University (Second Military Medical University), Shanghai, China.

3. Department of General Surgery, Naval Medical Center of People's Liberation Army (PLA), Shanghai, China.

**Corresponding author.**

Kailian Zheng, Department of Hepatobiliary Pancreatic Surgery, Changhai Hospital, Navy Medical University, NO. 168 changhai road, Yangpu District, Shanghai, 200433, China; Phone: +86 13818945716; Fax: +86 021-31161628; Email: [zhengkl828@126.com](mailto:zhengkl828@126.com); ORCID: 0000-0001-6195-3487

Gang Jin, Department of Hepatobiliary Pancreatic Surgery, Changhai Hospital, Navy Medical University, NO. 168 changhai road, Yangpu District, Shanghai, 200433, China; Phone: +86 021-31161629; Fax: +86 021-31161629; E-mail: [jingang@smmu.edu.cn](mailto:jingang@smmu.edu.cn); ORCID: 0000-0001-6713-1185

**Supplementary Materials - Index**

| **Supplementary Figures and Tables** |  |
| --- | --- |
| Table S1 | *pag. 2* |
|  |  |

**Supplementary Figures and Tables**

| **Table S1.** Risk Factor Analysis for ischemic complications. | | | | | |
| --- | --- | --- | --- | --- | --- |
|  | **Univariable** | | | **Multivariable** | |
| **Variable** | **Non-ischemic complication (n = 89)** | **Ischemic complication (n = 17)** | **P-value** | **Odds ratio**  **[95% CI]** | **P-value** |
| mAFA technique, n (%) | 33 (37.1) | 2 (11.8) | 0.042 | 0.006 (0.000-0.447) | 0.020 |
| Age, median (IQR, years) | 65.0 (56.0-69.0) | 63.0 (53.5-71.0) | 0.654 |  |  |
| Gender, n (%) |  |  | 0.612 |  |  |
| Male | 53 (59.6) | 9 (9.9) |  |  |  |
| Female | 36 (40.4) | 8 (47.1) |  |  |  |
| BMI, median (IQR, kg/m2) | 22.8 (20.2-24.4) | 23.0 (19.8-24.3) | 0.993 |  |  |
| Previous abdominal surgery, n (%) | 19 (21.3) | 2 (11.8) | 0.564 |  |  |
| Diabetes mellitus, n (%) | 20 (22.5) | 3 (17.6) | 0.904 |  |  |
| Coronary artery disease, n (%) | 6 (5.7) | 0 (0) | 0.569 |  |  |
| Hypertension, n (%) | 28 (31.5) | 6 (35.3) | 0.765 |  |  |
| Cerebrovascular disease, n (%) | 4 (4.5) | 0 (0) | 0.844 |  |  |
| Hepatic diseases, n (%) | 6 (5.7) | 0 (0) | 0.596 |  |  |
| Smoking history, n (%) | 23 (25.8) | 4 (23.5) | 1.000 |  |  |
| Drinking history, n (%) | 9 (10.1) | 2 (11.8) | 1.000 |  |  |
| Presenting symptoms, n (%) |  |  |  |  |  |
| Abdominal pain | 64 (71.9) | 11 (64.7) | 0.759 |  |  |
| Dyspepsia | 32 (36.0) | 5 (29.4) | 0.604 |  |  |
| Back pain | 40 (44.9) | 11 (64.7) | 0.135 |  |  |
| Weight loss | 41 (46.1) | 9 (52.9) | 0.603 |  |  |
| Medical examination detected | 10 (11.2) | 1 (5.9) | 0.819 |  |  |
| Neoadjuvant radiotherapy, n (%) | 9 (10.1) | 2 (11.8) | 1.000 |  |  |
| Neoadjuvant chemotherapy, n (%) | 26 (29.2) | 3 (17.6) | 0.494 |  |  |
| Preoperative CA 19-9, median (IQR, U/mL) | 165.6 (13.7-1168.9) | 477.0 (204.2-1200.0) | 0.031 | 1.000 (0.998-1.001) | 0.825 |
| Preoperative CEA, median (IQR, U/mL) | 3.32 (2.0-6.8) | 2.9 (1.9-5.4) | 0.431 |  |  |
| Operative time, min, median (IQR) | 135.0 (120.0-165.0) | 150.0 (106.0-180.0) | 0.775 |  |  |
| Blood loss, median (IQR, mL) | 500.0 (300.0-800.0) | 650.0 (425.0-800.0) | 0.193 |  |  |
| Blood transfusion, n (%) | 21 (23.6) | 3 (17.6) | 0.825 |  |  |
| Portal vein resection, n (%) | 13 (14.6) | 2 (11.8) | 1.000 |  |  |
| Left adrenalectomy, n (%) | 48 (53.9) | 11 (64.7) | 0.413 |  |  |
| Partial gastrectomy, n (%) | 5 (5.6) | 2 (11.8) | 0.688 |  |  |
| Tumour size, cm, median (IQR) | 4.0 (3.0-5.4) | 5.0 (4.3-6.0) | 0.014 | 1.498 (0.920-2.439) | 0.104 |
